# Antimicrobial potential of 27 plants consumed by chimpanzees (*Pan troglodytes verus* Blumenbach) in Ivory Coast

**DOI:** 10.1186/s12906-015-0918-7

**Published:** 2015-10-23

**Authors:** Angora Rémi Constant Ahoua, Amoin Georgette Konan, Bassirou Bonfoh, Mamidou Witabouna Koné

**Affiliations:** Unité de Formation et de Recherche des Sciences de la Nature, Université Nangui Abrogoua, BP 801 Abidjan 02 Abidjan, Côte d’Ivoire; Centre Suisse de Recherches Scientifiques en Côte d’Ivoire, BP 1303 Abidjan 01 Abidjan, Côte d’Ivoire; Laboratoire de Biochimie et Science des aliments, Unité de Formation et de Recherche des Biosciences, Université Félix Houphouët-Boigny, BP 582 Abidjan 22 Abidjan, Côte d’Ivoire

**Keywords:** Chimpanzee’s diet, Antimicrobial, Bacteria, Yeast, *Beilschmiedia mannii*, *Tristemma coronatum*, Ivory Coast

## Abstract

**Background:**

Due to their genetic proximity, chimpanzees share with human several diseases including bacterial, fungal and viral infections, such as candidiasis, acquired immune deficiency syndrome (AIDS), Ebola virus disease. However, in its natural environment, chimpanzees are tolerant to several pathogens including simian immunodeficiency virus (SIV), virus related to human immunodeficiency virus (HIV) that contribute to the emergence of opportunistic diseases such as microbial infections.

**Methods:**

Twenty seven species of plants consumed by chimpanzees were evaluated for their antimicrobial potential against *Escherichia coli*, *Pseudomonas aeruginosa, Staphylococcus aureus, Candida albicans, Candida tropicalis* and *Candida glabrata* using the agar diffusion technique and micro-dilution in 96-well plates. In total 132 extracts (33 dichloromethane, 33 methanol, 33 ethyl acetate and 33 aqueous) were tested.

**Results:**

The results showed that 24 extracts (18 %) showed activity against bacteria and 6 extracts (5 %) were active against yeasts. The minimal inhibitory concentrations (MICs) values of active extracts ranged between 23 and 750 μg/ml for bacteria and between 188 and 1500 μg/ml for yeasts.

**Conclusion:**

*Tristemma coronatum* was the most promising on the studied microorganisms followed by *Beilschmiedia mannii*. The extracts of the two plants indicated by chimpanzees have potential for antimicrobial use in human.

## Background

The use of plants in herbal medicine is very old and is experiencing a resurgence interest with the public [1]. According to World Health Organization (WHO), approximately, 75–80 % of the world’s population use plant medicine either in part or entirely for their health and care needs [2]. Chimpanzees are mainly frugivorous. This practice seems to have a beneficial effect on their health and well-being. Thus, it is worth exploring their behavioral trend to self-medicate in order to identify plants with therapeutic potential to fight certain human diseases including bacteria and fungi. These diseases are getting more common in humans with the development of drug resistance.

Infectious diseases caused by bacteria and fungi affect millions of people worldwide [3]. According to Soro et al. [4], bacterial and fungal infections, especially candidiasis, are among the most dangerous and deadly opportunistic diseases for vulnerable people such as children, the elderly and immunocompromised persons.

Their control becomes complex due to the emergence of multiresistant bacteria and fungi to many conventional antibiotics and antifungals [5–12]. Combined with the scarcity of new drugs on the market in recent years, this increase in bacterial and fungal resistance worldwide is a major threat to public health. According to the 2014 WHO report, in Africa, there is strong resistance of *Escherichia coli* to cephalosporins and third generation fluoroquinolones; two types of essential and widely used antibacterial drugs. It is also noted that 80 % of *Staphylococcus aureus* are resistant to methicillin (MRSA) in some parts of this region [13]. Also, there is an increased resistance to azoles such as fluconazole, the antifungal drug of choice in many countries, and the recently introduced antifungal agent, echinocandins [14]. In Ivory Coast, many cases of multidrug-resistant bacteria are reported [6–10]. According to Guessennd et al. [15], the frequency of bacterial strains resistant to imipenem increased from 1.9 % in 2005 to 5.9 % in 2009 and that of bacteria producing beta-lactamase with extended spectrum, from 5.3 % in 2005 to 16.8 % in 2009.

With the growing concern generated by the use of drugs, both in therapy and in the food industry, there has been in recent years an increase in interest in medicinal plants [16]. Many medicinal plants have shown their effectiveness against microbial infections across Africa, particularly in Ivory Coast [17–20]. However, the consumption pattern of chimpanzees, essentially composed of plant organs, remains poorly explored in this field. Chimpanzee remains morphologically and genetically the closest animal to humans, with 98 % of common DNA [21]. This genetic proximity expressed at many levels, including sharing with humans many zoonotic infections such as candidiasis and other bacterial infections. However, it is known that chimpanzees in their natural habitat are tolerant or resilient to SIV (Simian immunodeficiency virus) while humans are vulnerable to HIV, virus related to the simian virus [22, 23]. This tolerance or resilience of chimpanzee to SIV could be of genetic origin but also due to the environment. Their consumption pattern and feeding habit could indeed greatly contribute to their health, because in captivity, these animals develop many diseases [24]. It is recognized in pathophysiology that the presence of HIV in the body leads to a weakening of the immune system, promoting the emergence of opportunistic infections such as bacterial diseases and candidiasis [25].

A preliminary study carried out by Ahoua et al. [26] on the therapeutic potential, including the antioxidant capacity of eight plant species from Taï’s chimpanzee’s diet and harvested in the same area, but different from those investigated in this work, showed that these plants have strong antioxidant activity.

Based on the “One Health” concept [26–29], that promotes interdisciplinary and intersectorial collaborations at the human-animal-environment interface, the current study explores chimpanzees eating habits and behaviors towards self-medication to identify plants with strong antimicrobial potential able to play a role in the control of infections in humans. These plants could bring added value to the herbal medicine to solve health problems in humans. The main objective is to assess the antimicrobial potential of plants falling within the diet of chimpanzees in Tai National Park (Ivory Coast).

## Methods

### Selection of plant species

**Fig. 1:**
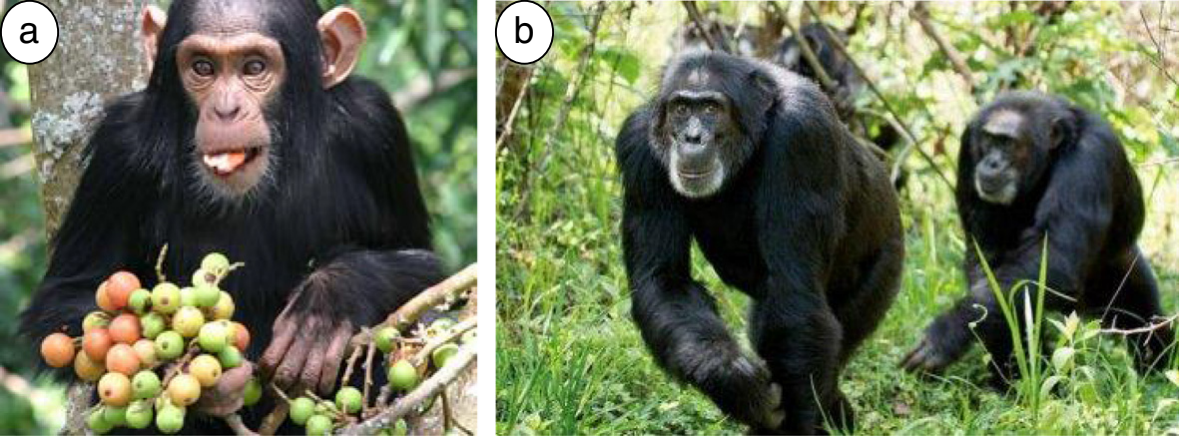
Chimpanzees (*Pan troglodytes verus*) in Taï National Park. **a** Young chimpanzee eating Ficus fruits (Source: http://pin.primate.wisc.edu/factsheets/french/chimpanzee); **b**-Group of chimpanzees (Source: http://www.sunservices.org/sun/index.php/tourisme/nos-parcs-et-reserves)

**Fig. 2:**
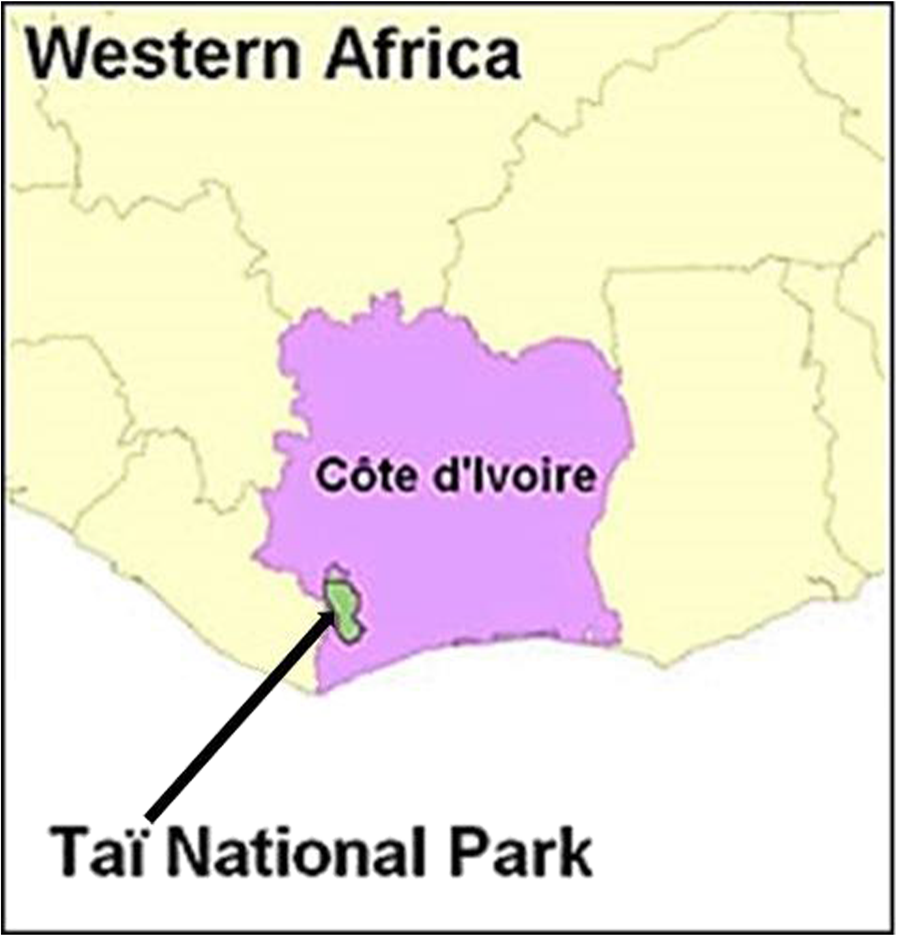
Localization of Taï National Park on Ivory Coast map (Source: wildchimps.org)

The plant species used for antimicrobial investigations came from the diet of chimpanzees (*Pan troglodytes verus*) (Fig. 1) from Taï National Park (TNP), located in the Southwestern region of Ivory Coast (Fig. 2). The research authorization was provided by the Ministry of Higher Education and Scientific Research of Ivory Coast while access and work in the park was issued by the Ministry of the Environment and Sustainable Development through the Ivorian Parks and Reserves Office.

**Fig. 3:**
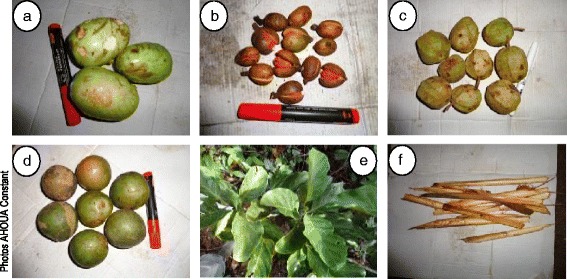
Example of plant organs consumed by chimpanzees. **a** *Magnistipula butayei* fruits; **b** *Pycnanthus angolensis* fruits; **c** *Duboscia viridiflora* fruits; **d** *Panda oleosa* fruits; **e** *Nauclea diderrichii* leaves; **f** *Ancistrophyllum secondiflorum* marrows

These plants were identified through direct observations in response to a previous study carried by N’Guessan [30] on Taï’s National Park chimpanzee’s diet. Many criteria including the consumption frequency of plant species, the consumed quantity and the duration of consumption allowed us to select 27 plant species among 131 reported. These plants were harvested in TNP in May and November 2012. The different parts harvested were leaves, fruits (pericarp, mesocarp and endocarp) and marrows (Fig. 3). Herbarium specimens were deposited in the herbarium of Swiss Centre of Scientific Research in Ivory Coast. Table 1 shows the different harvested plants.

**Table 1:** List of tested plant species

N°	Plant species	Family	Plant part used
1	*Afzelia bella* Harms	Leguminosae	Leaves
2	*Ancistrophyllum secundiflorum* (P.Beauv.) G.Mann & H.Wendl.	Arecaceae	Marrow
3	*Beilschmiedia mannii* (Meisn.) Benth. & Hook. f.	Lauraceae	Whole fruits; Pericarp; Mesocarp; Endocarp
4	*Calpocalyx aubrevillei* Pellegr.	Leguminosae	Leaves
5	*Chrysophyllum taiense* Aubrév. & Pellegr.	Sapotaceae	Leaves
6	*Coula edulis* Baill.	Olacaceae	Fruits (Endocarp)
7	*Dacryodes klaineana* (Pierre) H.J.Lam.	Burseraceae	Fruits
8	*Dialium aubrevillei* Pellegr.	Leguminosae	Fruits
9	*Dichapetalum pallidum* (Oliv.) Engl.	Dichapetalaceae	Leaves
10	*Dioscorea multiflora* Mart. ex Griseb.	Dioscoreaceae	Leaves
11	*Duboscia viridiflora* (K.Schum.) Mildbr.	Malvaceae	Fruits
12	*Glyphaea brevis* (Spreng.) Monach.	Tiliaceae	Leaves
13	*Halopegia azurea* (K.Schum.) K.Schum.	Marantaceae	Marrow
14	*Irvingia grandifolia* (Engl.) Engl.	Irvingiaceae	Fruits
15	*Keayodendron bridelioides* Gilg & Mildbr. ex Hutch. & Dalziel	Euphorbiaceae	Leaves
16	*Klainedoxa gabonensis* Pierre ex Engl.	Irvingiaceae	Fruits (Pericarp)
17	*Landolphia hirsuta* (Hua) Pichon	Apocynaceae	Leaves
18	*Magnistipula butayei* De Wild*.*	Chrysobalanaceae	Fruits (Pericarp and endocarp)
19	*Manniophyton fulvum* Müll. Arg.	Euphorbiaceae	Leaves
20	*Musanga cecropiodes* R. Br. ex Tedlie	Moraceae	Leaves
21	*Nauclea diderrichii* (De Wild.) Merr.	Rubiaceae	Fruits; Leaves
22	*Panda oleosa* Pierre	Pandaceae	Fruits (Pericarp)
23	*Parinari excelsa* Sabine	Chrysobalanaceae	Fruits (Pericarp and endocarp)
24	*Platysepalum hirsutum* (Dunn) Hepper	Leguminosae	Leaves
25	*Sarcophrynium brachystachys* (Benth.) K. Schum.	Marantaceae	Marrow
26	*Sterculia oblonga* Mast*.*	Sterculiaceae	Leaves
27	*Tristemma coronatum* Benth*.*	Melastomataceae	Leaves

### Extracts preparation

The samples were cleaned immediately after collection by washing them with water, dried under air at 18 °C for 3 weeks for the leaves and marrows. Fruits were lyophilized and then pulverized. Afterwards, 20 g of plant powder were macerated successively in 200 ml of organic solvent (dichloromethane, ethyl acetate and methanol) twice for 24 hours. Otherwise, 20 g of plant powder were macerated in 200 ml of water in the same conditions. The macerates obtained were then filtered and evaporated with a rotavapor at 40 °C. The methanol and aqueous extracts were then lyophilized. Dichloromethane and ethyl acetate extracts were evaporated to dryness.

### Assessment of the antimicrobial activity

#### Tested microorganisms

The antimicrobial activity was assessed against seven strains of bacteria including *Escherichia coli* ATCC 25922, *Escherichia coli* CIP 54127AF, *Pseudomonas aeruginosa* CIP 103467, *Pseudomonas aeruginosa* ATCC 27853, *Staphylococcus aureus* sensitive to penicillin, *Staphylococcus aureus* ATCC 25923, *Staphylococcus aureus* CIP 4.83 and four strains of yeast (*Candida albicans* (1), *Candida albicans* (2), *Candida tropicalis* and *Candida glabrata*). These reference and clinical strains were provided by the National Laboratory of Public Health of Ivory Coast and the Microbiology Laboratory of Swiss Centre of Scientific Research in Ivory Coast.

The antimicrobial activity was evaluated according to the protocol described by Koné et al. [18] using the agar diffusion technique. Stock solutions of plant extracts were prepared at 30 mg/ml in dimethyl sulfoxide (DMSO) and at 1 mg/ml (in distilled water) for antibiotics (tetracycline and gentamicin) and antifungals (nystatin and amphotericin B).

#### Antibacterial activity

##### Sensitivity test

**Fig. 4:**
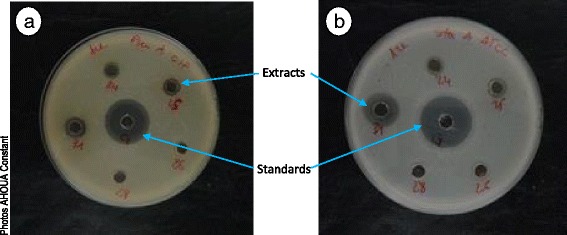
Inhibitory diameters of some ethyl acetate extracts on two bacteria *Pseudomonas aeruginosa* CIP 103467 (**a**) and *Staphylococcus aureus* ATCC 25923 (**b**) 24 = *Musanga cecropioides*; 25 = *Landolphia hirsuta*; 26 = Endocarp of *Beilschmiedia mannii* fruits; 28 = *Duboscia viridiflora*; 31 = *Klainedoxa gabonensis*; G = gentamicin

Mueller-Hinton agar in Petri dishes (thickness = 4 mm) were soaked with an inoculum equivalent to 0.5 of McFarland. After drying, wells (diameter = 6 mm) were made in the agar using sterile Pasteur pipette. Fifty microliters (50 μl) of extract (1.5 mg/ml) or antibiotic (0.025 mg/ml) was poured in the wells. Plates were left at ambient laboratory temperature for 15 to 30 min for a pre-diffusion of the solutions, and then incubated at 37 °C for 18 h. After incubation, the diameters (mm) of inhibition zones were measured (Fig. 4). The tests were carried out twice.

##### Determination of Minimum Inhibitory Concentration (MIC)

The extracts showing an inhibitory diameter of at least 10 mm were selected to determine the minimum inhibitory concentrations (MICs) using broth microdilution method in 96-wells microplates [18]. The MIC is the lowest concentration at which the visible growth of a strain was completely inhibited (no visible turbidity in wells). The plant extracts were solubilized in DMSO (30 mg/ml) and serially diluted in Mueller-Hinton medium, from 1500 to 1.5 μg/ml. The final concentrations were 25 to 0.05 μg/ml for antibiotics. All the tested bacteria were used with an initial inoculum of 3 × 10^6^ bacteria/ml. The microplates were incubated at 37 °C for 18 h.

##### Determination of Minimum Bactericidal Concentration (MBC)

MBC is the lowest concentration of antibiotic or crude extract in which less than 0.01 % of the initial inoculum survived after 18–24 h. Medium from wells with no visible growth and from the initial inoculum (dilution 10^−1^, 10^−2^, 10^−3^; 10^−4^ and 10^−5^) were plated on agar, and colonies counted. The value MBC/MIC allowed to determine whether an extract was bacteriostatic (MBC/MIC > 4) or bactericidal (MBC/MIC < 4) [31].

#### Antifungal activity

##### Sensitivity test

**Fig. 5:**
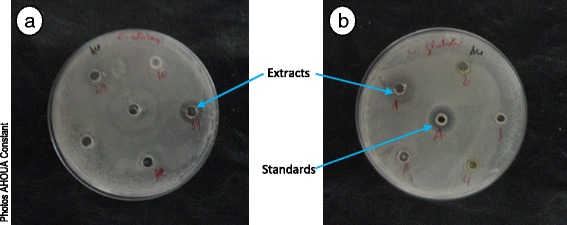
Inhibitory diameters of some ethyl acetate extracts on two yeasts *Candida albicans* ATCC 10231 (**a**) and *Candida glabrata* (**b**). 1 = *Dacryodes klaineana*; 2 = *Chrysophyllum taiense*; 3 = Pericarp of *Panda oleosa* fruits; 4 = *Dichapetalum pallidum*; 5 = Mesocarp of *Beilschmiedia mannii* fruits; 29 = Pericarp of *Beilschmiedia mannii* fruits; 30 = Endocarp of *Parinari excelsa* fruits; 31 = *Klainedoxa gabonensis*

Tryptone Soya Agar in Petri dishes was soaked with a yeast suspension. After drying, wells (diameter = 6 mm) were made in the agar using a sterile Pasteur pipette. A fraction (50 μl) of plant extract at 1.5 mg/ml or standard antifungal at 0.025 mg/ml was poured in the wells. Plates were left at ambient laboratory temperature for 15 to 30 min for a pre-diffusion of the solutions and then incubated at 30 °C for 48 to 72 h. After incubation, the diameters (mm) of inhibition zones were measured (Fig. 5). The tests were carried out twice for result validation.

##### Determination of Minimum Inhibition Concentration (MIC)

Extracts with inhibitory diameters greater than or equal to 10 mm, were selected to determine the minimum inhibitory concentrations (MICs) by using a microdilution method in liquid medium [16]. Plants extracts (1500 to 1.5 μg/ml) or nystatin and amphotericin B, positive controls (25 to 0.05 μg/ml) were serially diluted in Sabouraud broth in microplates (96 wells). This was followed by the addition of 50 μl of the inoculum to each well. The microplates were incubated at 30 °C for 48 to 72 h. After incubation, 40 μl of chloride methylthiazoyltetrazolium (MTT) at 0.2 mg/ml (in water) were added to each well, and further incubated again for 30 min at room temperature. The wells with no growth of yeast were colored in yellow and the lowest concentration (MIC) was determined [32]. The experiment was performed in duplicate in the same plate and tests were repeated twice.

### Statistical analysis

Microsoft Access 2007 was used for data entry. Data were then exported to Excel for analysis. The software STATISTICA 7.1 was used for data analysis [33]. Results were presented as mean ± SD of duplicate experiments. One-way analysis of variance (ANOVA) was used to compare extract’s inhibitory diameters. Significant differences between extracts were determined at *P* < 0.05. The least significant difference (LSD) between extracts was performed by the HDS test of Tukey.

## Results and discussion

The results of this study showed that out of the 132 tested extracts, 24 (18 %) were active against bacteria (Tables 2 and 3) and 6 (5 %) against fungi (Table 4). The inhibition zones diameters were between 10 and 20 mm. The MICs values ranged between 23 and 750 μg/ml for extracts and between 0.10 and 3 μg/ml for standards (0.10 to 3 μg/ml for gentamicin; 0.20 to 1.6 μg/ml for tetracycline). The active extracts were bactericidals and bacteriostatics (Table 3). A MIC value of 188 μg/ml or lower was considered a reasonable cut-off point for crude extracts.

**Table 2:** Inhibitory diameters (mm) of extracts on tested bacteria*

			Bacteria						
Plant species	Plant parts used	Extracts	*S. a.* CIP 4.83	*S. a.* Sen	*S. a.* ATCC 25923	*E.coli* CIP 54127AF	*E. coli* ATCC 25925	*P. a.* CIP 103467	*P. a.* ATCC 27853
*Beilschmiedia mannii*	Pericarp	DCM	15 ± 0.5^cd^	7 ± 0.5^lm^	0	0	0	9 ± 0.0^gj^	0
	Mesocarp	DCM	15 ± 1.0^cd^	14 ± 1.0^c^	13 ± 2.0^fg^	0	0	12 ± 1.5^cf^	0
		EtOAc	12 ± 0.5^e^	12 ± 0.5^de^	10 ± 0.0^hj^	0	0	13 ± 0.5^ce^	0
		MeOH	10 ± 0.5^fg^	11 ± 0.5^eg^	9 ± 0.5^jk^	0	0	10 ± 0.0^fi^	0
		Aqueous	0	10 ± 0.0^eh^	0	0	0	0	0
	Endocarp	DCM	0	9 ± 0.5^hk^	0	0	0	10 ± 0.0^fi^	0
	Whole fruits	EtOAc	10 ± 0.0^f^	11 ± 0.0^df^	9 ± 0.5^jk^	0	0	11 ± 0.5^eh^	0
		MeOH	10 ± 1.0^f^	11 ± 0.0^df^	11 ± 0.5^hi^	0	0	11 ± 0.0^eh^	0
*Chrysophyllum taiense*	Leaves	EtOAc	0	11 ± 0.5^eg^	0	0	0	11 ± 0.5^eh^	0
		MeOH	8 ± 0.5^ij^	10 ± 0.0^eh^	9 ± 0.0^ik^	0	0	10 ± 0.5^fj^	0
*Nauclea diderrichii*	Leaves	DCM	0	13 ± 0.5^cd^	0	0	0	14 ± 2.5^c^	0
		MeOH	10 ± 0.5^fg^	10 ± 0.0^eh^	9 ± 0.0^ik^	0	0	9 ± 0.5^fj^	0
*Manniophyton fulvum*	Leaves	MeOH	0	9 ± 0.50^hk^	15 ± 0.5^ef^	0	0	9 ± 0.5^hj^	0
*Calpocalyx aubrevillei*	Leaves	EtOAc	13 ± 0.5^e^	13 ± 0.5^cd^	12 ± 0.5^gh^	0	0	13 ± 0.0^cd^	0
		MeOH	9 ± 0.5^gi^	11 ± 0.5^eg^	9 ± 0.0^ik^	0	0	10 ± 1.0^fj^	0
*Dacryodes klaineana*	Fruits	EtOAc	0	0	18 ± 0.5^cd^	0	0	9 ± 1.5^gj^	0
*Landolphia hirsuta*	Leaves	MeOH	9 ± 0.5^gi^	10 ± 0.5^fi^	7 ± 0.5^l^	0	0	10 ± 0.5^fj^	0
*Platysepalum hirsutum*	Leaves	EtOAc	0	9 ± 0.5^fi^	0	0	0	10 ± 0.0^fi^	0
		MeOH	9 ± 0.5^gi^	10 ± 0.5^fi^	8 ± 0.5^kl^	0	0	10 ± 0.5^fj^	0
*Klainedoxa gabonensis*	Pericarp	EtOAc	15 ± 0.5^cd^	10 ± 0.0^eh^	16 ± 0.0^de^	0	0	11 ± 0.0^dg^	0
*Sterculia oblonga*	Leaves	DCM	0	11 ± 0.0^df^	0	0	0	0	0
*Magnistipula butayei*	Pericarp	DCM	15 ± 0.0^cd^	9 ± 0.0^gj^	9 ± 0.5^ijk^	0	0	9 ± 1.5^gj^	0
*Tristemma coronatum*	Leaves	EtOAc	16 ± 1.0^c^	9 ± 0.5^fi^	19 ± 1.0^c^	0	0	10 ± 0.5^fj^	0
		MeOH	0	9 ± 0.0^fi^	17 ± 1.5^d^	0	0	8 ± 0.0^ij^	0
Gentamicin			20 ± 0.5^b^	22 ± 1.5^a^	22 ± 0.5^b^	21 ± 2.5^a^	0	29 ± 1.0^a^	29 ± 1.5^a^
Tetracycline			23 ± 0.5^a^	19 ± 1.0^b^	30 ± 1.5^a^	17 ± 1.0^b^	0	21 ± 0.5^b^	24 ± 1.0^b^
F			432.11	268.08	290.02	97.082		176.88	423.89
P			< 0.001						

*: Mean ± SD of two replicates; DCM: Dichloromethane; EtOAc: Ethyl Acetate; MeOH: Methanol; *S. a.*: *Staphylococcus*; *E*: *Escherichia*; *P. a.*: *Pseudomonas aeruginosa*; ATCC: American Type Culture Collection; CIP: Centre Institut Pasteur; Sen: Sensitive; F: Fisher statistical; P: Probability; Values with the same superscript letter are not significantly different (*P* < 0.001)

**Table 3:** Antibacterial powers of active extracts

			Bacteria				
Plant species	Plant part used	Extracts		*S. a.* CIP 4.83	*S. a.* Sen	*S. a.* ATCC 25923	*P. a.* CIP 103467
*Chrysophyllum taiense*	Leaves	MeOH	MIC	>1500	188	>1500	188
			MBC		1500		750
			MBC/MIC		8		4
			APw		bacstat		bact
		EtOAc	MIC	>1500	188	>1500	188
			MBC		750		750
			MBC/MIC		4		4
			APw		bact		bact
*Manniophyton fulvum*	Leaves	MeOH	MIC	>1500	>1500	47	>1500
			MBC			1500	
			MBC/MIC			32	
			APw			bacstat	
*Calpocalyx aubrevillei*	Leaves	MeOH	MIC	>1500	94	>1500	94
			MBC		1500		750
			MBC/MIC		16		8
			APw		bacstat		bact
		EtOAc	MIC	188	23	23	47
			MBC	750	375	375	375
			MBC/MIC	4	16	16	8
			APw	bact	bacstat	bacstat	bacstat
*Beilschmiedia mannii*	Mesocarp	DCM	MIC	47	47	47	94
			MBC	47	94	1500	94
			MBC/MIC	1	2	32	1
			APw	bact	bact	bacstat	bac
		EtOAc	MIC	188	188	94	94
			MBC	>1500	375	375	375
			MBC/MIC	>8	2	4	4
			APw	bacstat	bact	bact	bact
		MeOH	MIC	94	188	>1500	188
			MBC	>1500	>1500		>1500
			MBC/MIC	>16	>8		>8
			APw	bacstat	bacstat		bacstat
		Aqueous	MIC	>1500	94	>1500	>1500
			MBC		>1500		
			MBC/MIC		>16		
			APw		bacstat		
*Platysepalum hirsutum*	Leaves	MeOH	MIC	>1500	375	>1500	375
			MBC		>1500		1500
			MBC/MIC		>4		4
			APw		bacstat		bact
		EtOAc	MIC	>1500	>1500	>1500	187
			MBC				750
			MBC/MIC				4
			APw				bact
*Landolphia hirsuta*	Leaves	MeOH	MIC	>1500	375	>1500	375
			MBC		1500		750
			MBC/MIC		4		2
			APw		bact		bact
*Beilschmiedia mannii*	Whole fruits	MeOH	MIC	94	375	94	188
			MBC	375	750	>1500	750
			MBC/MIC	4	2	>16	4
			APw	bact	bact	bacstat	bact
		EtOAc	MIC	188	47	>1500	188
			MBC	>1500	750		1500
			MBC/MIC	>8	16		8
			APw	bacstat	bacstat		bacstat
*Nauclea diderrichii*	Leaves	DCM	MIC	>1500	375	>1500	375
			MBC		1500		750
			MBC/MIC		4		2
			APw		bact		bact
		MeOH	MIC	94	188	>1500	>1500
			MBC	750	750		
			MBC/MIC	8	4		
			APw	bacstat	bact		
*Tristemma coronatum*	Leaves	EtOAc	MIC	188	>1500	47	188
			MBC	>1500		1500	750
			MBC/MIC	>8		32	4
			APw	bacstat		bacstat	bact
		MeOH	MIC	>1500	>1500	23	>1500
			MBC			375	
			MBC/MIC			16	
			APw			bacstat	
*Beilschmedia mannii*	Endocarp	DCM	MIC	>1500	>1500	>1500	750
			MBC				>1500
			MBC/MIC				>2
			APw				bacstat
*Beilschmedia mannii*	Pericarp	DCM	MIC	94	>1500	>1500	>1500
			MBC	750			
			MBC/MIC	8			
			APw	bacstat			
*Sterculia oblonga*	Leaves	DCM	MIC	>1500	94	>1500	>1500
			MBC		1500		
			MBC/MIC		16		
			APw		bacstat		
*Magnistipula butayei*	Pericarp	DCM	MIC	94	>1500	>1500	>1500
			MBC	750			
			MBC/MIC	8			
			APw	bacstat			
*Dacryodes klaineana*	Fruits	EtOAc	MIC	>1500	>1500	23	>1500
			MBC			750	
			MBC/MIC			33	
			APw			bacstat	
*Klainedoxa gabonensis*	Pericarp	EtOAc	MIC	188	94	47	188
			MBC	1500	750	750	750
			MBC/MIC	8	8	16	4
			APw	bacstat	bacstat	bacstat	bact
Gentamicin			MIC	0.10	0.8	0.10	3
			MBC	0.4	0.8	0.10	3
			MBC/MIC	4	1	1	1
			APw	bact	bact	bact	bact
Tetracycline			MIC	0.8	0.2	0.4	1.6
			MBC	6	6	0.4	3
			MBC/MIC	8	30	1	2
			APw	bacstat	bacstat	bact	bact

*MIC* minimal inhibitory concentration (μg/ml); *CMB* minimum bactericidal concentration (μg/ml); *APw* antibacterial power; *Bacstat* bacteriostatic; *Bact* bactericidal; *S. a. Staphylococcus*; *P. a. Pseudomonas aeruginosa; ATCC* American type culture collection; *CIP* Centre Institut Pasteur; *Sen* sensitive

**Table 4:** Inhibitory diameters (mm) of active extracts on *Candida* strains*

			Fungi			
Plant species	Plant part used	Extracts	*C. albicans* (1)	*C. albicans* (2)	*C. tropicalis*	*C. glabrata*
*Ancistrophyllum secundiflorum*	Marrow	DCM	0	0	0	0
		EtOAc	0	0	0	0
		MeOH	0	0	0	11 ± 0.5^c^
		Aqueous	0	0	0	0
*Manniophyton fulvum*	Leaves	DCM	0	0	0	0
		EtOAc	0	0	0	0
		MeOH	12 ± 0.0^b^	0	0	0
		Aqueous	0	0	0	0
*Dacryodes klaineana*	Fruits	DCM	0	0	0	0
		EtOAc	12 ± 0.5^bc^	6 ± 0.0^c^	0	15 ± 0.5^a^
		MeOH	0	0	0	0
		Aqueous	0	0	0	0
*Klainedoxa gabonensis*	Pericarp	DCM	0	0	0	0
		EtOAc	12 ± 0.0^b^	8 ± 0.5^a^	10 ± 0.0^c^	10 ± 0.0^c^
		MeOH	0	0	0	0
		Aqueous	0	0	0	0
*Tristemma coronatum*	Leaves	DCM	0	0	0	0
		EtOAc	12 ± 0.0^b^	0	0	13 ± 0.5^b^
		MeOH	12 ± 0.5^bc^	0	11 ± 0.0^b^	12 ± 0.0^b^
		Aqueous	0	0	0	0
Amphotericin B			14 ± 0.0^a^	0	16 ± 0.5^a^	15 ± 0.0^a^
Nystatine			11 ± 1.0^c^	0	11 ± 1.0^b^	13 ± 0.5^b^
F			495.94	993.87	404.49	1091.4
P			< 0.001			

*: Mean ± SD of two replicates; DCM: Dichloromethane; EtOAc: Ethyl Acetate; MeOH: Methanol; C: *Candida*; F: Fisher statistical; P: Probability; Values with the same superscript letter are not significantly different (*P* < 0.001)

In short, eight extracts were the most active showing MICs below this limit. These extracts showed activities against more than two strains of bacteria with low MIC values, namely the ethyl acetate extract of the leaves from *C. aubrevillei* (23–188 μg/ml), the ethyl acetate and dichloromethane extracts of the fruits mesocarp from *B. mannii* (94–188 μg/ml and 47–94 μg/ml, respectively), the ethyl acetate extract of the fruits pericarp from *K. gabonensis* (47–188 μg/ml) and the methanol extract of the whole fruits from *B. mannii* (94–375 μg/ml). These extracts were followed by those with the methanol extract of the fruits mesocarp from *B. mannii* (94–188 μg/ml) and the ethyl acetate extracts from the whole fruits of *B. mannii* (47–188 μg/ml) and the leaves of *T. coronatum* (47–188 μg/ml)*.* For most of these plants, mainly *B. mannii* and *C. aubrevillei*, it is the first time that their antimicrobial activity is demonstrated. However, *B. mannii* was previously studied for other activities. Thus, the fruits of *B. mannii* were reported to have low antioxidant property [34]. Moreover, antibacterial activity of related species of *B. mannii* was screened. So, Fankam et al. [35] showed that the methanol extract of fruits from *Beilschmiedia obscura* exhibited antimicrobial activity on various bacterial strains including *P. aeruginosa*, a multiresistant bacteria. Compounds isolated from the stem bark of *Beilschmiedia anacardioides* showed activity against *Bacillus subtilis, Micrococcus luteus* and *Streptococcus faecalis* [36]. Phytochemical studies of several species of *Beilschmiedia* genus revealed the presence of antibacterial compounds such as alkaloids [37], flavonoids [38, 39] and phenolics, and related compounds [40]. These compounds could explain the bactericidal activity of *B. mannii* where inhibitory diameters (10–15 mm) found with the extracts were the closest to those of gentamicin (20–29 mm) and tetracycline (17–30 mm)*.* The activities observed in various species of *Beilschmiedia* showed that this genus has the potential to fight against bacteria and could be exploited to search for potential antibacterial molecules.

Concerning *C. aubrevillei*, no biological and phytochemical studies were reported on this species. However, it was reported that this plant is also consumed by Bossou chimpanzees in Guinea [41]. On the opposite, in the western region of Ivory Coast, it fits in the diet of people [42]. According to the results obtained in this study, this plant has mainly bacteriostatic effects with inhibitory diameters mainly around 13 mm and MICs values between 23 and 188 μg/ml.

Nevertheless, none of the tested extracts showed activity against the two strains of *E. coli* and that of *P. aeruginosa* ATCC 27853. Gentamicin and tetracycline showed activities (17–30 mm) on all strains.

The methanol extract of the leaves from *T. coronatum* and the ethyl acetate extracts from *K. gabonensis* fruits showed activity against *C. tropicalis*, *C. albicans* (1) and *C. glabrata* (Table 4)*.* The ethyl acetate extract of the leaves from *T. coronatum* and the fruits from *D. klaineana* were effective against *C. albicans* (1) and *C. glabrata* while the methanolic extracts of the leaves from *M. fulvum* and the marrow of *Ancistrophyllum secundiflorum* were only active against *C. albicans* (1) and *C. glabrata*, respectively. Amphotericin B and nystatin did not show any activity against *C. albicans* (2), while the ethyl acetate extracts from *D. klaineana* fruits and the fruits pericarp of *K. gabonensis* showed low activity in this strain with respectively 6 and 8 mm inhibitory diameter. The MICs values of the active extracts ranged between 188 and 1500 μg/ml and were greater or equal to 25 μg/ml for the positive controls (>25 μg/ml for amphotericin B; ≥ 25 μg/ml for nystatin) (Table 5). The methanol and ethyl acetate extracts of the leaves from *T. coronatum* as well as the ethyl acetate extract from the pericarp of *K. gabonensis* were the most active.

**Table 5:** Minimal Inhibitory Concentration (MIC) of active extracts on yeasts

			MIC (μg/ml)		
Plant species	Plant part used	Extracts	*C. albicans* (1)	*C. tropicalis*	*C. glabrata*
*Manniophyton fulvum*	Leaves	MeOH	1500	nd	nd
*Tristemma coronatum*	Leaves	EtOAc	750	nd	375
		MeOH	>1500	375	375
*Ancistrophyllum secundiflorum*	Marrow	MeOH	nd	nd	>1500
*Dacryodes klaineana*	Fruits	EtOAc	188	nd	nd
*Klainedoxa gabonensis*	Pericarp	EtOAc	750	188	750
Amphotericin B			>25	>25	>25
Nystatine			25	>25	>25

*EtOAc* ethyl acetate, *MeOH* methanol, *C Candida*, *nd* non determined

The ethyl acetate extract from *K. gabonensis* fruits and from the leaves of *T. coronatum* showed an activity against fungi as well as, bacteria and were mainly bacteriostatics. These two plant extracts showed the greatest inhibitory diameters on bacteria. For *T. coronatum* it is also the first time, to our knowledge, that the antimicrobial activity is reported. This plant showed the greatest inhibitory diameters specifically on *S. aureus* CIP 4.83 (16 mm) and *S. aureus* ATCC 25923 (17–19 mm) and also yeasts (11–13 mm) with MICs values between 375 and 1500 μg/ml. *K. gabonensis* was already studied for its antimicrobial properties. Wansi et al. [43] showed the effect of some compounds isolated from the stem bark of this plant, in particular ellagic acid, 3,3′-dimethyl ether ellagic acid, gallic acid and the methyl gallate on various sources of microbes including *E. coli, S. aureus* and *C. albicans.* These compounds could be the basis of the bacteriostatic effects or the antifungal activities (MIC between 188 and 750 μg/ml) of the studied fruits in this work. In Cameroon, *K. gabonensis* is used to treat various diseases including bacterial diseases [44]. On the opposite, in Congo, it is used by traditional healers for the treatment of dermatitis incurable by modern medicine [45]. In Ivory Coast, any indication of the therapeutic use of this plant species has not yet been reported. Therefore, the results obtained in this study with the fruits of this plant confirm the traditional uses already described above.

## Conclusion

The results of this study showed that the plants consumed by chimpanzees of TNP possess strong antimicrobial activity. The use of plants by chimpanzees confirms the self-medication in the natural setting. These plants are poorly exploited in various areas, including their antimicrobial potential. Faced with the resistance of microorganisms to various conventional drugs, it would be interesting to explore the feeding behavior of primates in order to make profits. These plants could help to fight efficiently against several pathologies especially opportunistic diseases. Interestingly, promising plants such as *T. coronatum* and *B. mannii* were identified. The extracts of those plants could be used for further investigation in human medicine. We plan to investigate other biological activities of these species and their phytochemicals.

## Abbreviations

DCM: Dichloromethane
EtOAc: Ethyl acetate
MeOH: Methanol
*S. a.*: *Staphylococcus*
*E*: *Escherichia*
*P. a.*: *Pseudomonas aeruginosa*
C: *Candida*
ATCC: American type culture collection
CIP: Centre Institut Pasteur
Sen: Sensitive
F: Fisher statistical
P: Probability
MIC: Minimal inhibitory concentration (μg/ml)
CMB: Minimum bactericidal concentration (μg/ml)
APw: Antibacterial power
Bacstat: Bacteriostatic
Bact: Bactericidal
